# Torque movement of the upper anterior teeth using a clear aligner in cases of extraction: a finite element study

**DOI:** 10.1186/s40510-022-00421-8

**Published:** 2022-08-01

**Authors:** Yuxun Cheng, Jie Gao, Shishu Fang, Wei Wang, Yanning Ma, Zuolin Jin

**Affiliations:** 1 State Key Laboratory of Military Stomatology and National Clinical Research Center for Oral Diseases and Shaanxi Clinical Research Center for Oral Diseases, Department of Orthodontics, School of Stomatology, Air Force Medical University, Xi’an, 710032 China; 2 Urumql DW Innovation InfoTech Co., Ltd, Xinjiang, 830000 China; 3 Shanxi Medical University School and Hospital of Stomatology, Taiyuan, 030001 China

**Keywords:** Clear aligner, Torque, Anterior teeth, Tooth extraction, Finite element

## Abstract

**Background:**

Clear aligner treatment has become popular over recent years. It is necessary to identify methods by which we could avoid the bowing effect in extractions with clear aligner. The present study was to identify the appropriate method to design torque movement involving the upper anterior teeth of extraction cases, in order to maintain or improve the axis and torque of the upper anterior teeth with a clear aligner during movement and closure of the extraction space.

**Results:**

As the height of the power ridge increased, the rotation angle of the upper central incisor in the sagittal direction decreased gradually and the location of the rotation center changed significantly; the rotation center moved in the apical direction and then changed to the crown side. The highest von-Mises stress of the upper central incisor root, periodontal ligaments, and alveolar bone, showed little change as the power ridge height increased. When the axial inclination of the upper central incisor was normal (U1-SN = 105°), the tendency of movement for the upper central incisor approached translation with a power ridge height of 0.7 mm (corresponding distorted angle: 5.8415). When the axial inclination of the upper central incisor was oversized (U1-SN = 110°), the axial inclination of the upper central incisor reduced to normal following completion of the anterior segment retraction with a power ridge of 0.4 mm (corresponding distorted angle: 3.4265).

**Conclusion:**

Analysis indicates that pure palatal tipping movement of the upper anterior teeth is generated without torque control, thus resulting in the bowing effect. The required torque control of the upper anterior teeth with oversize axial inclination is weaker than that of the upper anterior teeth with normal axial inclination because limited torque loss is expected for oversize axial inclination teeth**.** Variation sensitivity of the rotation center should be considered carefully due to biological problems when designing translation of the upper anterior teeth with normal axial inclination.

## Background

Clear aligner treatment has become popular over recent years because it is both comfortable and aesthetically acceptable. On the one hand, this form of treatment involves a series of orthodontic operations covering all of the teeth and the keratinized gingiva without obvious damage to the periodontal tissues [1]. On the other hand, this treatment involves a form of tooth movement that features a predetermined “mismatch” between the aligner and the tooth. The force produced by the deformation of the appliance is transmitted to the tooth and periodontal tissues, thus causing tooth movement and tissue remodeling [2]. Over recent years, plastic aligners have been employed for mild to moderate orthodontic tooth movements [3]. However, when it comes to complicated cases, such as extractions, there are several biomechanical and biological problems associated with this treatment. For example, the bowing effect may arise in the process of anterior teeth retraction in extraction cases if an inappropriate strength is designed. The bowing effect can result in deep overbite of the anterior teeth, the open bite of premolars, and the mesial tipping of molars [4]. Researchers have attempted to identify methods by which we could avoid this side effect. For example, Lai demonstrated that appropriate aligner overtreatment with canine attachments should be designed to ensure bodily retraction of incisors in extraction cases [5]. Nevertheless, it remains unclear as to how to design and set up the torque on the anterior teeth in an appropriate manner. The bowing effect occurs because the appliance loses control of the tooth. The moment to force ratio (M/F ratio) can be used to control the patterns of tooth movement [6]. Therefore, it is reasonable to design torque movement by controlling the M/F ratio.

Using a mathematical model derived from the computer-aided design (CAD) discretization of solids in three dimensions, finite element analysis (FEA) can estimate the stresses generated within different tissues, such as alveolar bone, the PDL, and teeth, and can also determine the loading and displacement patterns of all structures [7]. Recent years, FEM has been widely applied in different dental fields, from fixtures to the simulation of dental movements to assess the stresses generated within the different tissue structures, such as alveolar bone, periodontal ligament, and teeth [8–10]. Therefore, it can contribute to our biomechanical understanding of clear aligner.

The aim of this paper was to use FEA to identify an appropriate technique to design torque movement when the upper anterior teeth were retracted in order to maintain or improve the axis and torque of the upper anterior teeth when undergoing movement caused by closure of the extraction space. Our overall goal is to provide direction for the use of clear aligners in the clinic.

## Methods

**Fig. 1:**
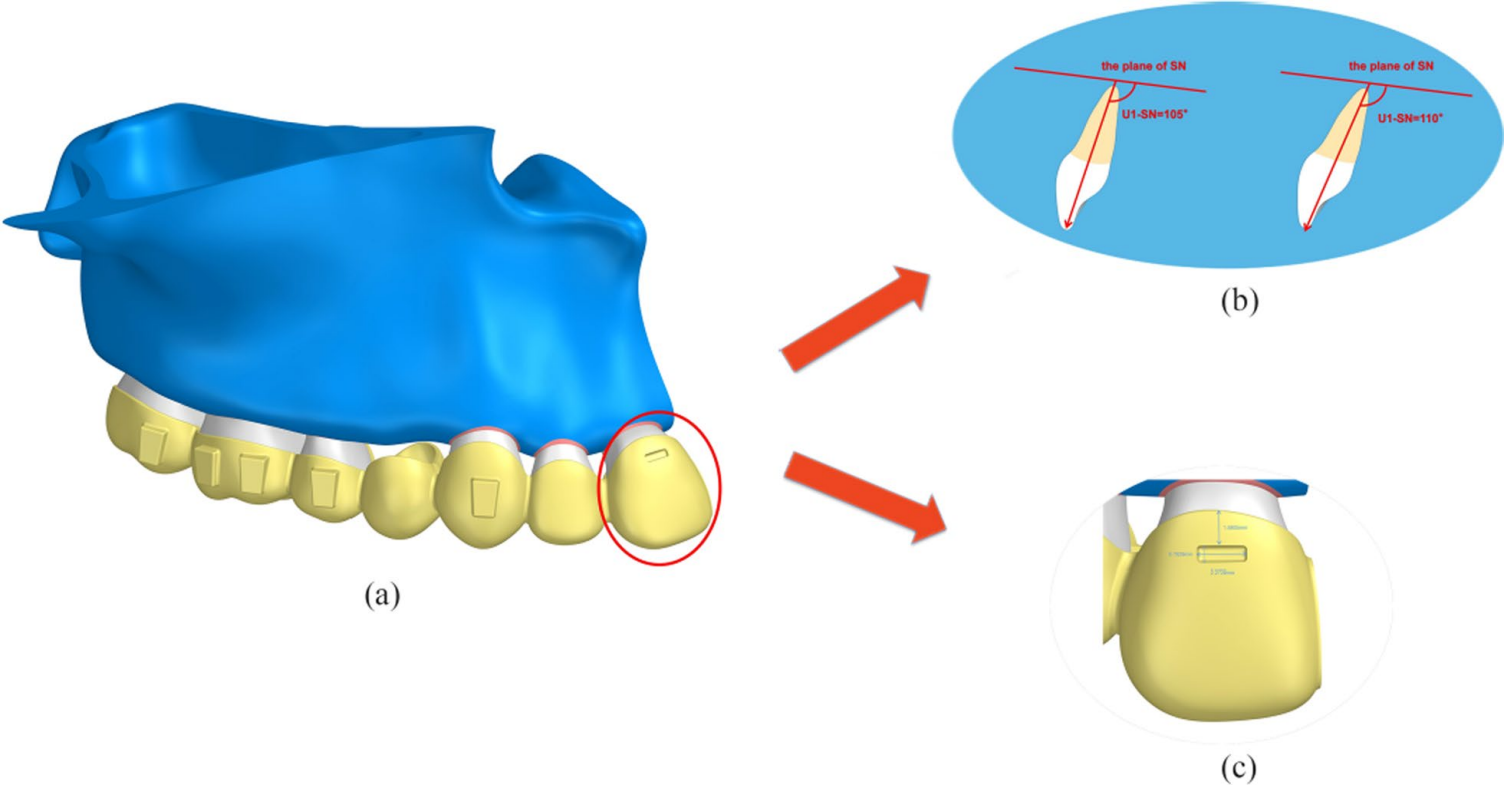
Three-dimensional finite element model for incisor retraction in a case involving 1st premolar extraction. **a** Maxillary arch with 1st premolar removal with attachments on the vestibular surfaces of the crowns and a geometric model of the clear aligner. **b** Different axial inclinations of the upper central incisor (U1-SN = 105°; U1-SN = 110°). **c** The position and size of the power ridge

Cone beam computed tomography (CBCT) data (HiRes3D-Plus; Largev, Beijing, China) were acquired from a healthy volunteer with well-aligned dentition and normal axial inclination of the upper anterior teeth. As shown in Fig. 1a, three dimensions base models of the maxillary dentition with extracted first premolar were established using MIMICS 20.0 (Materialise, Leuven, Belgium). GEOMAGIC Studio 2014 (Raindrop GEOMAGIC, North Carolina, USA) was used to optimize the basic model and create a surface model structure. With the help of NX1911 software (Siemens, German), the outer surface of the maxillary teeth roots was extended outward by 0.25 mm to generate a preliminary model for PDLs. The maxillary was also moved inward by 1.3 mm to generate a bone cortex and cancellous structure. Vertical rectangular attachments (3 mm height, 2 mm width, and 1 mm thickness) were designed for all teeth other than the central incisor and the lateral incisor. The maxillary crown and attachment were extended outward by 0.5 mm to simulate the thickness of the appliance, and each tooth was treated as an independent component. All components were imported into ANSYS Workbench 2019 (Ansys, Pennsylvania, USA) to generate a 3D finite element model for finite element analysis.

All structures were assumed as linear elastic isotropic and homogeneous materials. Their mechanical properties were obtained from previous studies [2, 11] and are shown in Table 1. It was worthy of note that most scholars still agreed that it was reasonable to study tooth movement tendency even if the PDL was assigned linear material properties in FEA [7, 12, 13]. Bonding was established at the following interfaces: ligament bone, tooth ligament, and tooth attachments. No separation conditions were constructed among tooth interfaces. Frictionless conditions were established on contact interfaces between the aligner and tooth crown surfaces and attachments. The establishment of frictionless conditions created a nonlinear connection; this meant that this was a nonlinear study.

**Table 1:** Properties of the materials considered in this study

Material	Young’s modulus, MPa	Poisson ratio
Alveolar bone	1.37 × 10^3^	0.3
Tooth	1.96 × 10^4^	0.3
PDL	6.9 × 10^−1^	0.45
Aligner	816	0.36
Attachment	12.5 × 10^3^	0.36

### Configuration settings

Two protocols were established according to the axial inclination of the upper central incisor (configuration 1: U1-SN = 105°; configuration 2: U1-SN = 110°) (Fig. 1b).

### Loading method

In this FE simulation of a clinical event, a retraction of 0.25 mm in the sagittal direction was imposed on the initial anterior region; this drove deformation of the aligner segment. The forces generated by the aligner on each tooth were then calculated by the software (ANSYS Workbench 15.0) and were then loaded back on the corresponding tooth in the reverse direction [2].

### Experimental design

For both two protocols, a displacement of 0.25 mm in the distal direction was preset for the maxillary anterior teeth to simulate one stage of anterior teeth retraction in the clinical setting. Different power ridge heights (0–1 mm) were used as a carrier to mimic torque control in the clinical setting. The upper central incisor was the main observation object of this study; therefore, the power ridge was set on the surface of the upper central incisor. The location and dimension of the power ridge are shown in Fig. 1c.

When the axial inclination was normal (105°), a group with a power ridge of 0 mm in height was taken as the control group, and power ridges of different heights (0–1 mm) were added on the basis of the control group until the rotation angle of the upper central incisor was close to 0° in the sagittal plane and the corresponding mechanics could favor translation. When the axial inclination was oversized (110°), the group with a power ridge of 0 mm in height was taken as the control group and power ridges of different heights (0–1 mm) were added on the basis of the control group until the oversize axial inclination of the upper central incisor to the Sella-Nasion (SN) plane reduced to normal after the completion of anterior retraction. That is, the rotation angle of the upper central incisor was limited to 5° in the sagittal direction. Assuming that the number of orthodontic steps was 60, the rotation angle of the upper central incisor in the sagittal direction was limited to 0.08333° per step.

## Results

### Control group

**Fig. 2:**
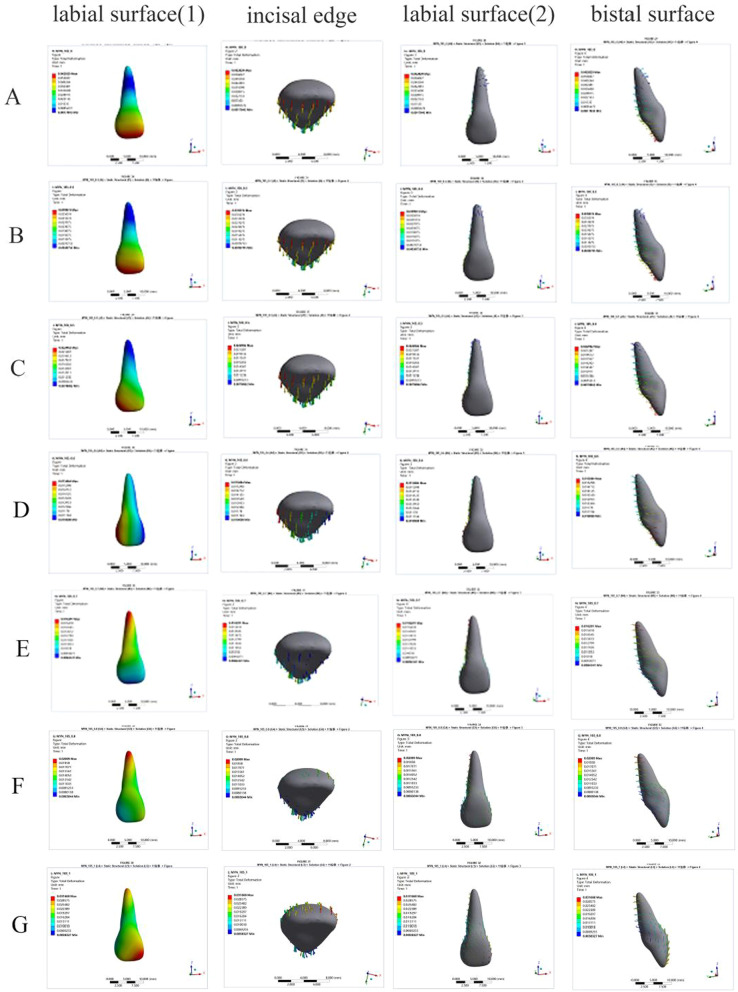
The total displacement trend of 11 when U1-SN = 105°. **A** The total displacement trend of 11 with the torque ridge height of 0 mm. **B** The total displacement trend of 11 with the torque ridge height of 0.3 mm. **C** The total displacement trend of 11 with the torque ridge height of 0.5 mm. **D** The total displacement trend of 11 with the torque ridge height of 0.6 mm. **E** The total displacement trend of 11 with the torque ridge height of 0.7 mm. **F** The total displacement trend of 11 with the torque ridge height of 0.8 mm. **G** The total displacement trend of 11 with the torque ridge height of 1.0 mm

**Fig. 3:**
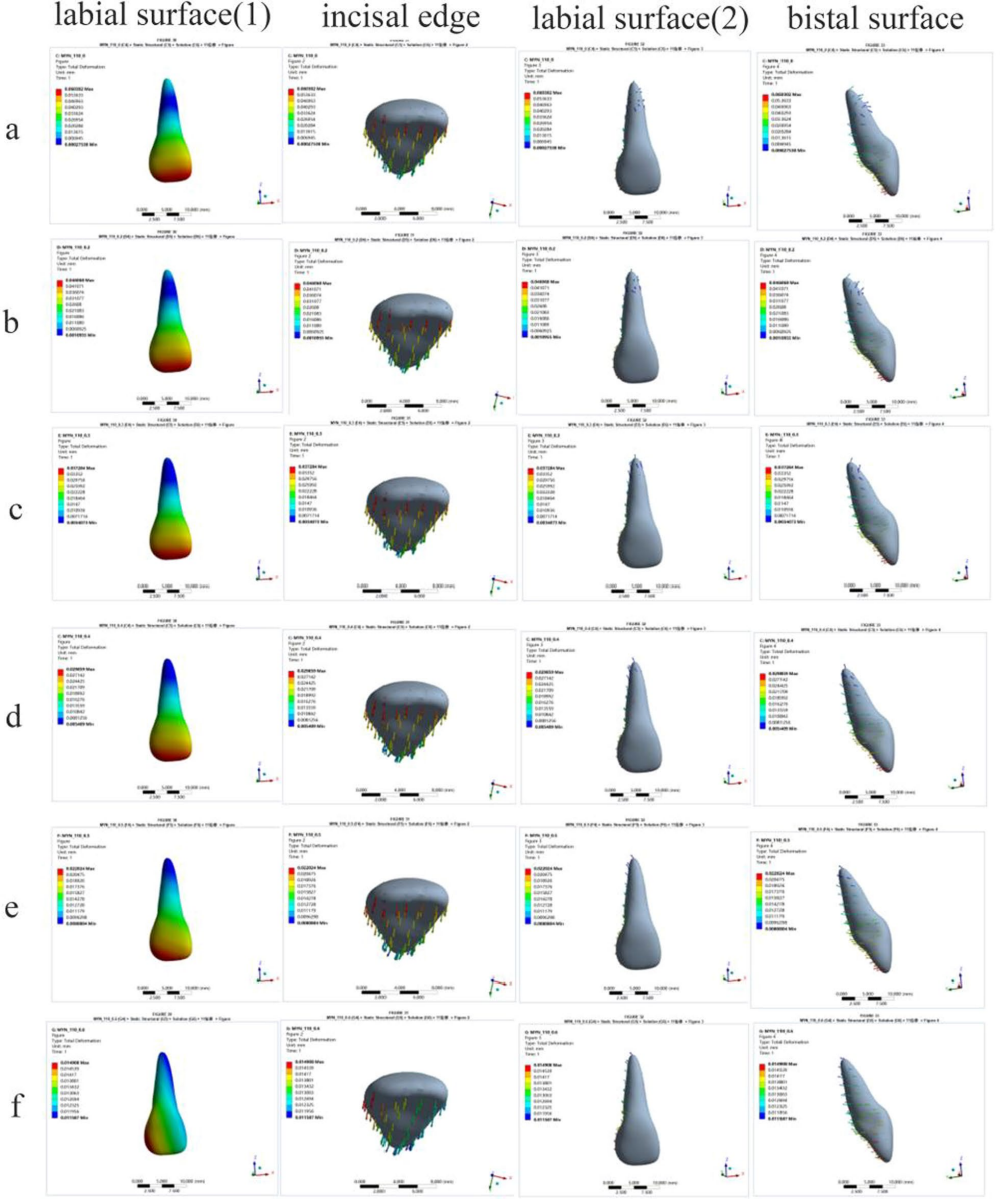
The total displacement trend of 11 when U1-SN = 110°. **A** The total displacement trend of 11 with the torque ridge height of 0 mm. **B** The total displacement trend of 11 with the torque ridge height of 0.2 mm. **C** The total displacement trend of 11 with the torque ridge height of 0.3 mm. **D** The total displacement trend of 11 with the torque ridge height of 0.4 mm. **E** The total displacement trend of 11 with the torque ridge height of 0.5 mm. **F** The total displacement trend of 11 with the torque ridge height of 0.6 mm

**Fig. 4:**
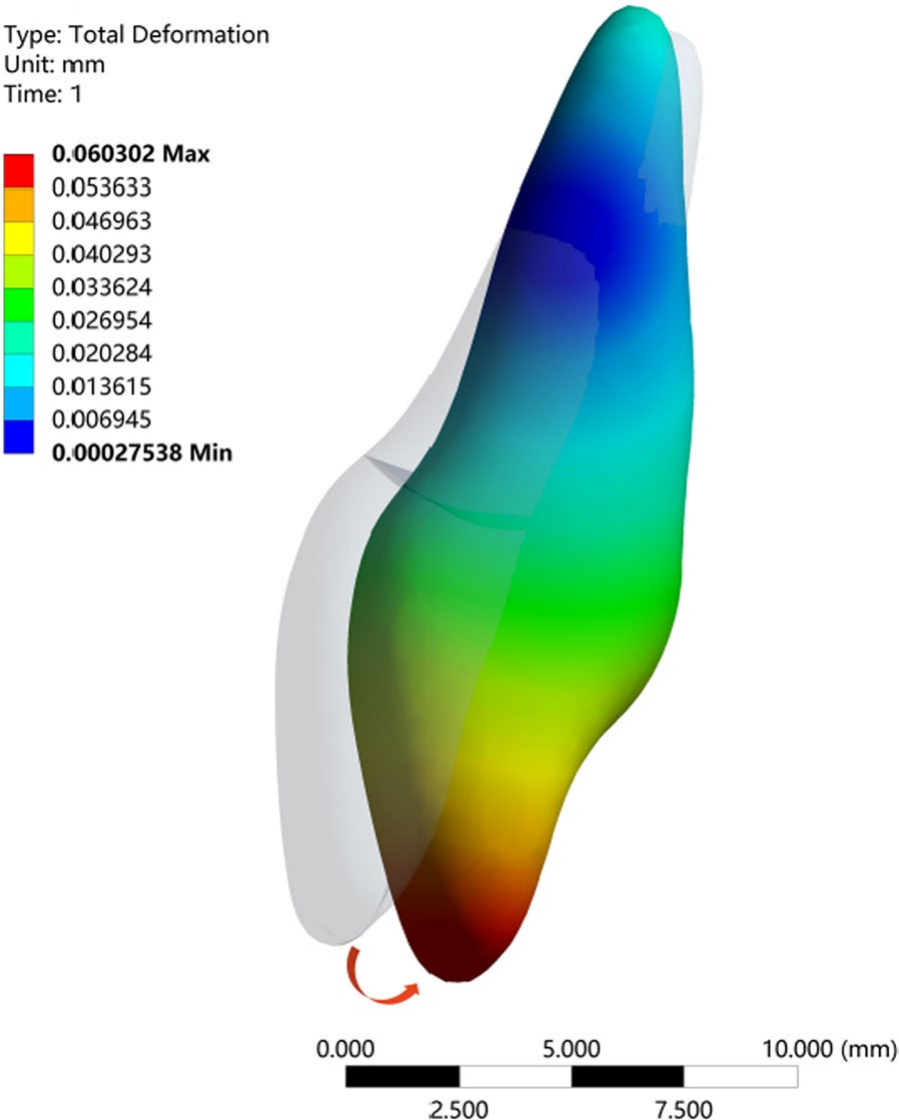
The clockwise moment and palatal inclination magnified by 50-fold for a tooth without a power ridge

In this study, we used an FE model to simulate retraction of the upper anterior teeth. The group with a power ridge of 0 mm in height was taken as the control group. When the axial inclination of the upper central incisor was 105°, the rotation center of the upper central incisor was located approximately between the middle and apical thirds of the root (Fig. 2a) and the rotation angle in the sagittal direction was 0.1688°. The highest von Mises stress values for different structures were 0.1567 MPa for the root, 0.0281 MPa for the PDL, and 0.5563 MPa for the alveolar bone. When the axial inclination of the upper central incisor was oversized (110°), the rotation center of the upper central incisor was still located between the middle and apical thirds of the root (Fig. 3a) and the rotation angle in the sagittal direction was 0.1630°. The highest von Mises stress values for different structures were 0.1391 MPa for the root, 0.0212 MPa for the PDL, and 0.5268 MPa for the alveolar bone. Generally, the upper central incisor showed a tendency for oblique movement without a power ridge (Fig. 4).

### The effect of different power ridge heights on the rotation center of the upper central incisor in the sagittal direction

Next, power ridges of different heights were added on the basis of the control group to simulate torque control (moment adding). Figure 2 shows the trends for change in the upper central incisor’s rotation center with an axial inclination of 105°, which gradually moved to the apical direction as the power ridge height increased from 0 to 0.5 mm; this became the same side as the crown when the height of the power ridge was 0.7 mm, 0.8 mm, and 1.0 mm. Moreover, the upper central incisor twisted in the distal direction when the power ridge height was 0.6 mm, thus indicating that the rotation center had changed significantly. However, when the axial inclination was 110°, the upper central incisor’s rotation center gradually moved in the apical direction as the height of the power ridge increased from 0 to 0.5 mm; the upper central incisor twisted in the distal direction when the power ridge height was 0.6 mm (Fig. 3).

### The effect of different power ridge heights on the rotation angle of the upper central incisor in the sagittal direction

**Fig. 5:**
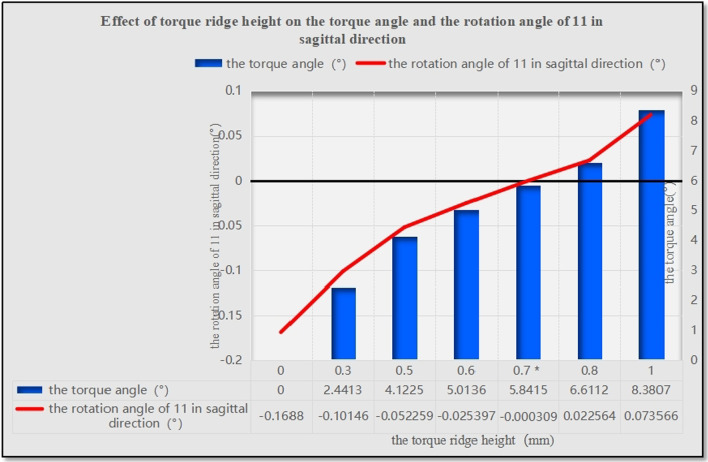
The rotation angle of 11 in the sagittal direction with different power ridge heights when the axial inclination of 11 was 105°. *When the power ridge height is 0.7 mm, the rotation angle of 11 was close to 0° and the displacement trend of 11 was most close to bodily movement

The rotation angle gradually decreased as the height of the power ridge was increased when the axial inclination of the upper central incisor was 105° (Fig. 5). It was notable that when the power ridge height was 0.6 mm, 0.7 mm, and 0.8 mm, the rotation angle of the upper central incisor was close to 0° (− 0.0254°, − 0.0030°, and 0.02256°, respectively). Furthermore, the upper central incisor twisted in the distal direction when the power ridge height was 0.6 mm (Fig. 2); when the height of the power ridge was 0.7 mm, the range of total displacement was 0.0078569 mm; this was only half the value when the height of the power ridge was 0.8 mm (0.0136 mm). Therefore, it was reasonable to consider that the displacement trend of the upper central incisor was most close to bodily movement when the power ridge height was 0.7 mm.

**Fig. 6:**
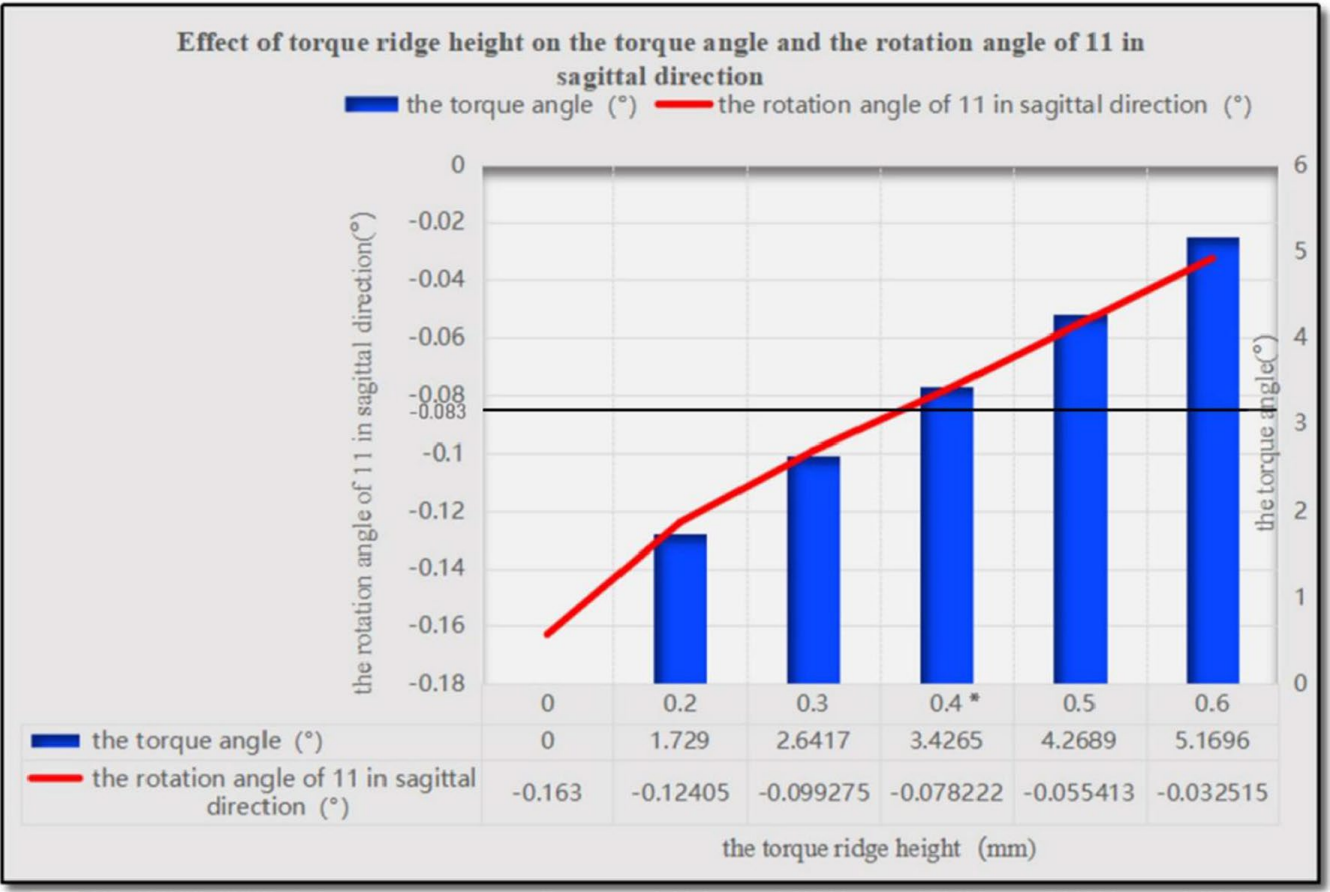
The rotation angle of 11 in the sagittal direction with different power ridge heights when the axial inclination of 11 was 110°. *When the height of the power ridge was 0.4 mm, the rotation angle of the upper central incisor was 0.078222° in the sagittal plane, which was very close to 0.08333°. That is, the axial inclination of an upper central incisor would decrease to 105° after the completion of anterior retraction

When the axial inclination of the upper central incisor was 110°, the changing trends for the rotation angles with different power ridge heights were similar to those with an axial inclination of 105° (Fig. 6). We considered that the axial inclination of an upper central incisor would decrease to 105° after the completion of anterior retraction. Therefore, it was necessary to limit the rotation angle of the upper central incisor to 5° in the sagittal direction. Considering that the number of orthodontic steps was 60, the rotation angle of the upper central incisor in the sagittal direction was limited to 0.08333° per step. When the height of the power ridge was 0.4 mm, the rotation angle of the upper central incisor was 0.078222° in the sagittal plane, which was very close to 0.08333° (Fig. 6).

### The effect of different torque ridge heights on the highest von-Mises stress values of the root, PDL, and alveolar bone

**Fig. 7:**
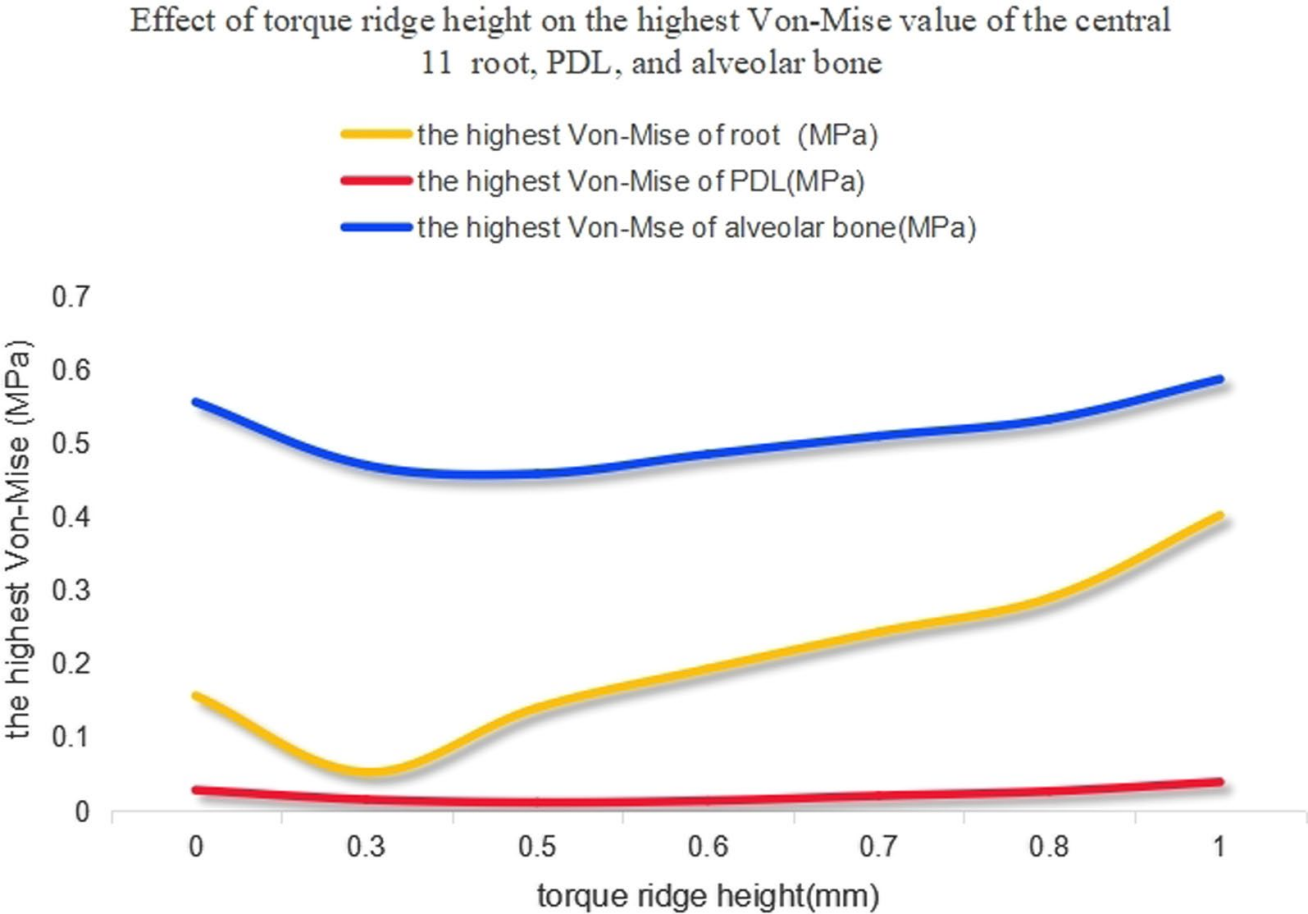
The highest von-Mises value of the central incisor root, PDL, and alveolar bone with different power ridge heights when U1-SN = 105°

**Fig. 8:**
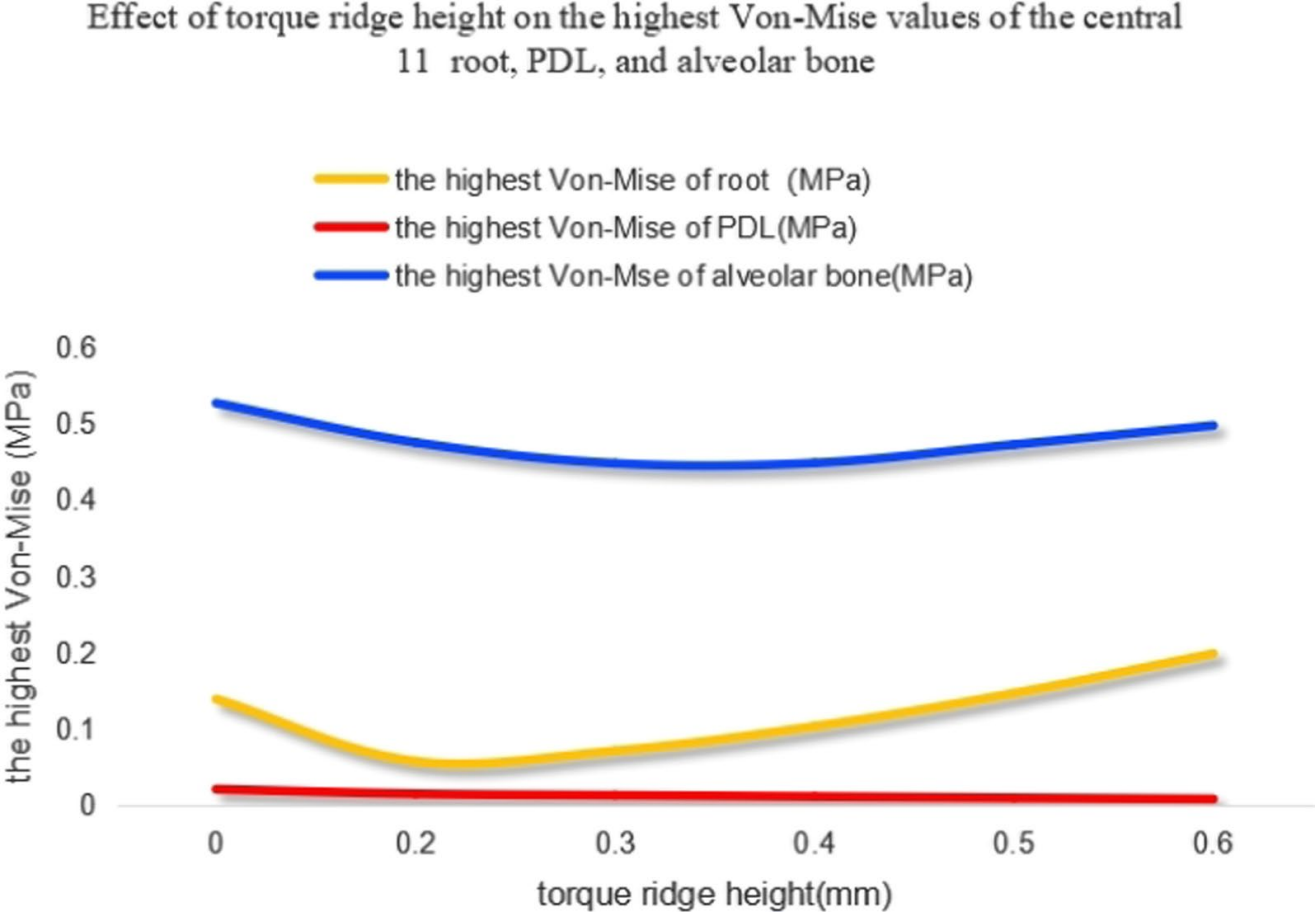
The highest von-Mises value of the central incisor root, PDL, and alveolar bone with different power ridge heights when U1-SN = 110°

When the axial inclination was 105°, when compared with the control group, the highest von-Mises stress value of the PDL and alveolar bone showed very little change as the height of the power ridge increased. Furthermore, compared with the control group, although the highest von-Mises stress value of the upper central incisor root showed an upward trend as the height of the power ridge was increased, they were the same order of magnitude (Fig. 7). When the axial inclination was 110°, the tendency for change in these structures was similar to those with an axial inclination of 105° (Fig. 8). Therefore, we considered that the highest von-Mises stress values of the root, PDL, and alveolar bone, in this study, were still within the normal physiological range.

## Discussion

FEA is a widely used and effective method to explore the biomechanical principles on orthodontic studies. Most FEA studies relate to the initial stress distribution and displacement pattern within different structures, including teeth and periodontal tissue [2, 14, 15]. Cortona et al. [15] used FEM to evaluate the influence of different staging and attachment configurations on the orthodontic rotational movement of a lower second premolar obtained with clear aligners. Ma et al. [2] establish the FE models to explore the optimal orthodontic displacement of clear aligner for patients with periodontitis. Given the application of FEM in oral biomechanics and orthodontics, the appropriate technique to design torque movement involving the anterior teeth of extraction cases with clear aligner was explored by FEM in the present study.

Previous studies determined that tipping movement is the most predictable movements with clear aligner, but there still remains somewhat difficult to achieve a comparable amount of root control [16–19]. Elkholy et al. [17] used 3D sensor to record the force to moment (F/M) induced by aligners for sagittal displacement and found that the bodily components of tooth movement generated by the aligners were negligibly small. Hahn et al. [18] suggested that aligners tended to ‘lift up’ in relation to the intended amount of root movement during torquing, and therefore no effective force couple could be established for further root control. These studies pointed out that pure tipping movement of the anterior teeth would be generated without additional torque compensation. This conclusion was confirmed by the phenomenon observed in the present study that the crown of the central incisor moved toward the palatal direction and the root apical segment moved toward the labial direction with the rotation center being located approximately between the middle and apical thirds of the root (Fig. 4).

Pertinent studies suggested that the force couple generated at the cervical and incisal regions of the aligners is not large enough to generate an adequate counter moment because of limitations in material properties [17, 20]. This situation could be eventually improved by selective modifications to aligners by means of mechanical reinforcement of the cervical area [17], such as the power ridge of Invisalign system. Simon found that the power ridge generated significant torque compensation (7.9 N.mm) during torque movement of upper central incisors, which confirmed the efficiency of power ridge in anterior teeth torque [21]. However, there were few studies focusing on the magnitude of the torque compensation for anterior teeth of different axial inclination.

**Fig. 9:**
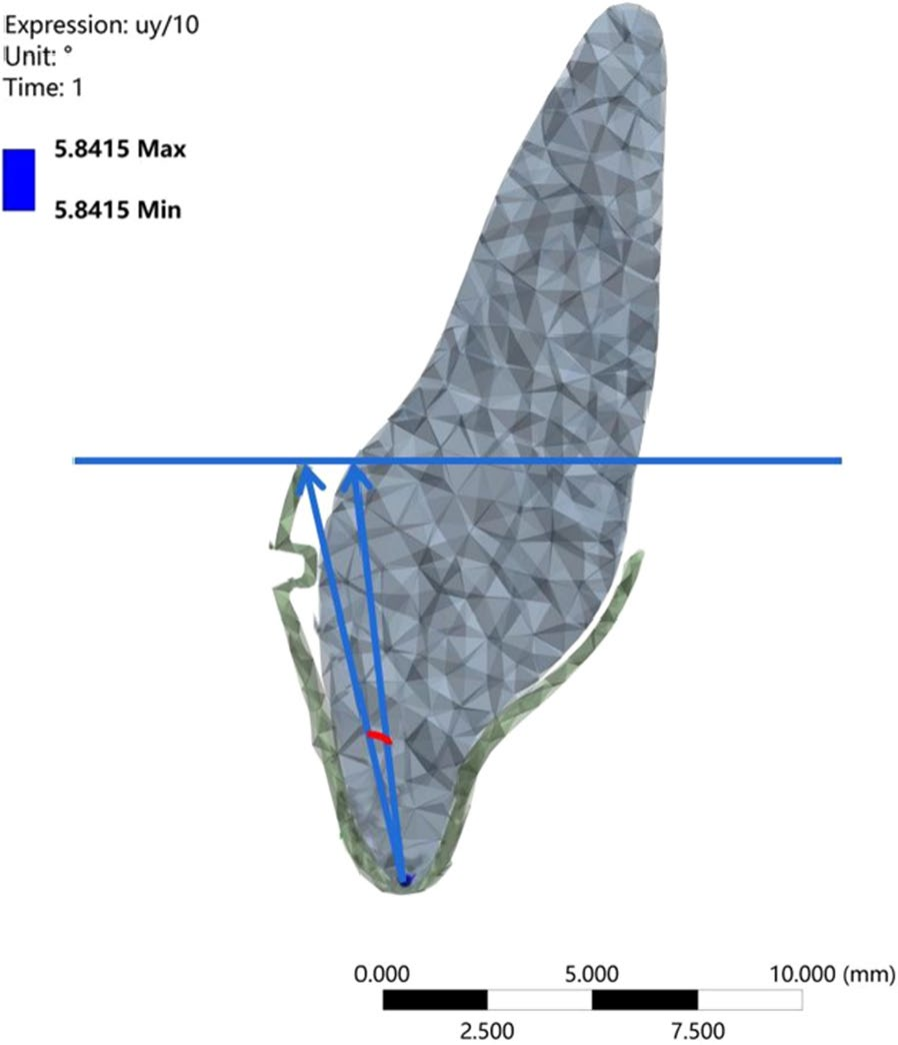
The angle of distortion between the inner surface of the aligner and the tooth surface generated by the power ridge

In essence, the presence of a power ridge in the tooth neck made the aligner undergo further distortion, thus generating an angle between the inner face of the aligner and the tooth surface, thereby favoring a counter moment (Fig. 9). From mechanical knowledge, this angle was determined by the height of the power ridge and reflected the magnitude of torque control; these parameters were correlated. That is, translation could be achieved with a distortion angle of 5.8415° (with a corresponding power ridge height of 0.7 mm), and torque movement could be achieved with a distortion angle of 3.4265° (with a corresponding power ridge height of 0.4 mm). Therefore, the required torque control for upper anterior teeth with oversize axial inclination was weaker than that of upper anterior teeth with normal axial inclination.

Another issue addressed in our study was the variation sensitivity of the CRot. The relative position between the center of resistance (CR) and CRot reflected the type of tooth movement. The CR is equal to the center of mass, which is not influenced by the M/F ratio. The method of finding CR of teeth has been explored by relevant studies in recent years. In a previous study, CR was determined by a second force that resulted in bodily tooth movement [22]. A subsequent study used a finite element approach to locate the CR in maxillary teeth [23]. In another paper, Christiansen et al. determined the CRot experimentally by applying different forces on the maxillary upper central incisors and confirmed that the CRot was related to the M/F ratio [24]. In this study, when the axial inclination of upper central incisor was normal, it could be considered that the CR-CRot distance increased until it became infinite (the CRot moved in the apical direction) when the height of the power ridge increased from 0 to 0.7 mm; then, the CRot changed to the crown side of the CR as the height of the power ridge increased. These findings concurred with previous studies in that the tendency of the CRot to change with the value of M/F was similar to inverse proportional function [25]. In particular, a slight change in the M/F load led to a large change in the CR-CRot distance as it approached bodily movement [25]. Therefore, variation sensitivity of the CRot should be considered carefully when managing biological problems in the clinical setting.

## Limitations

FEA represents one of the best ways to analyze force systems delivered by clear aligners. This study provided valuable information for the design of torque movement for upper anterior teeth with clear aligners in extraction cases. However, our findings must be clinically validated to support plastic aligners with solid evidence. One of the limitations of the present study was that FEA could not take the biological events into consideration, such as root length, root morphology, aligner material property, and so on. Another limitation was that the current FE model lied in the difficulty associated with simulating the large displacements on the tooth as a result of the long-term application of orthodontic forces. That was, FEA ignored the events of resorption–apposition present in variable time frames. What’s more, in the clinical scenario, an air gap between an aligner and a dentition is filled with saliva, causing friction between the aligner and the dentition. However, the friction was not designed in this model. Considering the limitations above, there was necessity of relevant clinical practice. Additionally, this study did not consider the unintended intrusion of the anterior teeth caused by distortion of the appliance. This was another side effect of extraction and needed to be considered carefully in further research.

## Conclusions

In this study, a pure palatal tipping movement of the anterior teeth is generated without torque control, thus resulting in the bowing effect. The required torque control for anterior teeth with oversize axial inclination is weaker than that of anterior teeth with normal axial inclination; this is because oversize axial inclination teeth need a limited loss of torque. Finally, the variation sensitivity of the CRot should be considered carefully in the clinical setting when designing translation of the upper anterior teeth with normal axial inclination. These conclusions provide direction for the use of clear aligners in the clinic.

## Data Availability

The datasets used and/or analyzed during the current study are available from the corresponding author on reasonable request.

## Abbreviations

FEMs: Finite element models
CRot: The rotation center
PDL: Periodontal ligaments
M/F ratio: The moment to force ratio
CAD: Computer-aided design
CBCT: Cone beam computed tomography
SN: Sella-Nasion
CR: The center of resistance
